# Evolutionary analysis of mitochondrially encoded proteins of toad-headed lizards, *Phrynocephalus,* along an altitudinal gradient

**DOI:** 10.1186/s12864-018-4569-1

**Published:** 2018-03-06

**Authors:** Yuanting Jin, Yubin Wo, Haojie Tong, Sen Song, Lixun Zhang, Richard P. Brown

**Affiliations:** 1 Zhejiang Provincial Key Laboratory of Biometrology and Inspection & Quarantine, College of Life Sciences, China Jiliang University, Hangzhou, 310018 People’s Republic of China; 2 School of Life Sciences, Lanzhou University, Lanzhou, 730000 People’s Republic of China; 3 School of Natural Sciences and Psychology, Liverpool John Moores University, Liverpool, L3 3AF UK

**Keywords:** *Phrynocephalus*, Mitochondrial genes, Complex I, Selective evolution, Altitude

## Abstract

**Background:**

Animals living at high altitude must adapt to environments with hypoxia and low temperatures, but relatively little is known about underlying genetic changes. Toad-headed lizards of the genus *Phrynocephalus* cover a broad altitudinal gradient of over 4000 m and are useful models for studies of such adaptive responses. In one of the first studies to have considered selection on mitochondrial protein-coding regions in an ectothermic group distributed over such a wide range of environments, we analysed nineteen complete mitochondrial genomes from all Chinese *Phrynocephalus* (including eight genomes sequenced for the first time). Initial analyses used site and branch-site model (program: PAML) approaches to examine nonsynonymous: synonymous substitution rates across the mtDNA tree.

**Results:**

Ten positively selected sites were discovered, nine of which corresponded to subunits *ND2*, *ND3*, *ND4*, *ND5*, and *ND6* within the respiratory chain enzyme mitochondrial Complex I (NADH Coenzyme Q oxidoreductase). Four of these sites showed evidence of general long-term selection across the group while the remainder showed evidence of episodic selection across different branches of the tree. Some of these branches corresponded to increases in altitude and/or latitude. Analyses of physicochemical changes in protein structures revealed that residue changes at sites that were under selection corresponded to major functional differences. Analyses of coevolution point to coevolution of selected sites within the *ND4* subunit, with key sites associated with proton translocation across the mitochondrial membrane.

**Conclusions:**

Our results identify mitochondrial Complex I as a target for environment-mediated selection in this group of lizards, a complex that frequently appears to be under selection in other organisms. This makes these lizards good candidates for more detailed future studies of molecular evolution.

**Electronic supplementary material:**

The online version of this article (10.1186/s12864-018-4569-1) contains supplementary material, which is available to authorized users.

## Background

Altitudinal gradients can produce environmental variation that has a major influence on the survival and evolution of organisms [1]. Animal populations commonly found at extremely high altitudes are expected to adapt their phenotypes, including physiology and metabolism, to local environmental conditions. Changes in the structure and function of proteins associated with these adaptive responses are therefore predicted.

The metabolic impacts of low temperature and hypoxia at high altitude suggest that proteins encoded by the mitochondrial genome could be integral to adaptive responses due to their association with oxidative phosphorylation of ADP to ATP. Five protein complexes participate in this process, which produces 95% of the energy used by eukaryotes [2]. Four of these complexes (I, III, IV, and V) contain polypeptides encoded by mitochondrial genes. Several studies have reported evidence of positive Darwinian selection on mitochondrial proteins, although purifying selection is more commonly detected [3, 4].

Previous studies of the effects of selection on mitochondrial evolution have primarily focused on mammals [5–13] and/or focused on single genes or complexes that are expected to have experienced positive selection [14, 15]. Nonetheless, some research has linked altitudinal/latitudinal gradients to selection-mediated mitochondrial evolution. For example, adaptive responses to northern latitudes or high altitudes have been linked to the evolution of mitochondrial-encoded proteins in modern humans [16–18]. Previous mammalian studies have also examined possible altitude-related selection on genes in the oxidative phosphorylation pathway. Positive selection on three amino acid residues in the ND2 and ND6 genes were found for one of two high altitude Chinese *Rhinopithecus* monkeys [17]**,** while analyses of the Caprini, which are adapted to a high altitude existence, detected greatest selection on ATPase and cytochrome b genes, and least selection on ND and COX genes [19]. Nevertheless, there do not seem to have been any studies of ectotherms occupying a wide altitudinal range to date.

The altitudinal range of Chinese *Phrynocephalus* lizards spans over 4200 m, with some species such as *P. theobaldi* inhabiting regions up to approximately 5000 m. The latter represents one of the world’s highest altitude lizard species. Viviparous *Phrynocephalus* are restricted to the Qinghai-Tibetan Plateau (QTP), while oviparous *Phrynocephalus* are only found at lower altitudes outside the QTP. Viviparous and oviparous Chinese *Phrynocephalus* split approximately 10 Ma ago, with subsequent diversification occurring at very different altitudes [20]. Different viviparous species can also occupy very different environments, e.g., *P. erythrurus* and *P. forsythii* which show a 4200 m altitudinal difference. Intraspecific altitudinal ranges can also be substantial, exceeding 1500 m in some cases [21].

The distributions of Chinese *Phrynocephalus* make them excellent models for the study of ectotherm adaptation to cold environments. Previous studies have highlighted the impact of altitude on geographic variation of intra- and interspecific life history traits in *Phrynocephalus* [21–24], as well as its influence on physiological and metabolic processes [25]. Comparative transcriptome analyses of *P. przewalskii* (oviparous) and *P. vlangalii* (viviparous) have detected several nuclear genes that are likely to have experienced selection as part of the shift to a high-altitude existence [26]. To date, no research has investigated altitude-related evolution of mitochondrial genes in *Phrynocephalus*, despite the importance of these genes for metabolic processes that could be affected by the low temperatures and oxygen concentrations found at high altitudes.

In this study, we examined mitochondrial protein evolution among all species and major evolutionary lineages within *Phrynocephalus* with the aim of: (1) detecting genes and sites that have experienced significant selection during the diversification of the group*,* (2) analysing whether selected sites have important functions and/or have undergone significant co-evolution with other key functional sites.

## Methods

### Mitochondrial genomes

All experimental protocols were performed in accordance with guidelines from the China Council on Animal Care and approved by the Ethics Committee for Animal Experiments at China Jiliang University.

**Fig. 1:**
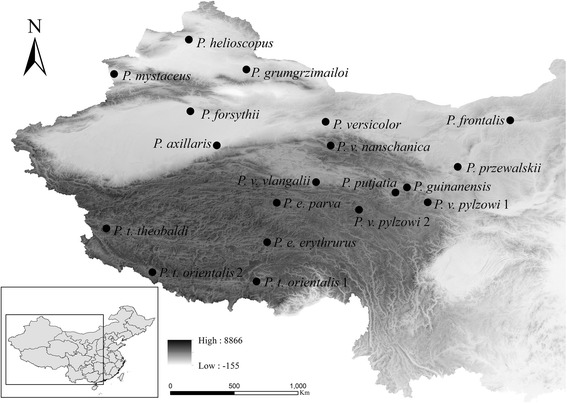
Map showing sites from which *Phrynocephalus* were sampled. All species/specimen numbers shown on the map correspond to Additional file 1 (map data were provided by the Scientific Data Centre of the Chinese Academy of Sciences and the final map produced by the authors using the software ArcGis 10.2)

A total of 19 complete mitogenomes representing all Chinese *Phrynocephalus* species were analysed in our study. The genomes provided a comprehensive coverage of deep mitochondrial lineages in the group. Seven of these mitogenomes have been previously published by our group (GenBank: KJ551842, KJ885621, KJ830752, KJ749841, KJ630904, KF572032, KM093859), and four mitogenomes had been published by other researchers (GenBank KC119493, KC578685, KM093858, KP126516). The origins of all samples are shown in Fig. 1 and more detailed information is provided in the Additional file 1.

Eight mitochondrial genomes represented by individuals primarily from Tibetan plateau were newly-sequenced for this study (GenBank MF039058–65) from tissues of voucher specimens stored at China Jiliang University and Lanzhou University. These mitogenomes represented the species/subspecies *P. vlangalii vlangalii*, *P. v. pylzowi*, *P. v. nanschanica*, *P. theobaldi theobaldi*, *P. t. orientalis*, *P. erythrurus erythrurus*, and *P. frontalis* were obtained using PCR. LA Taq Hot Start Version Polymerase (TaKaRa Bio, Osaka, Japan) was used with four pairs of designed primers (Additional file 2) and each portion was sequenced using a primer-walking method. The TA-rich region of the Control Region, which is difficult to sequence directly, was amplified from genomic DNA using primers containing a 5′ EcoRI and a 3′ X-hol site, cloned into the pGex-4 t-1 vector and sequenced. Full details of the mitogenome sequencing protocol are available in our previous work [27].

### Phylogenetic tree construction

Phylogenetic relationships among all of the taxa have been inferred by previous studies, however, given the availability of such a large mitochondrial dataset, we applied a maximum likelihood (ML) approach to re-examine these relationships and ensure a robust topology for analyses of selection. The genomes were divided into six partitions: the three codon positions, rRNA, tRNA and control region sequences. Small areas of potentially ambiguous alignment were trimmed from the rRNAs, tRNAs and control regions leaving a 15,422 bp alignment. Control region sequence was missing for *P. axillaris*. ML analyses were carried out using RAxML ver. v8.2.10 [28] on a Linux Debian operating system. The raxmlHPC-PTHREADS command was used for the analyses and the GTR + G model was specified for each partition (−m command). Oviparous and viviparous species are unequivocally monophyletic [20] and this was specified as a topological constraint (backbone option). An ML tree was identified for the original sequence and additional trees were determined for 2000 bootstrap-resampled datasets to indicate levels of support for branches (−# option). No outgroup was specified and so the ML tree was rooted with a midpoint root for display purposes.

### Tests of selection

Statistical analyses of selection are based on nonsynonymous: synonymous ratios (ω) within protein-coding sequence, across the (unrooted) mtDNA tree that we inferred here. We used the CODEML program in PAML ver. 4.8 [29], to detect signatures of selection using both more general site models and also branch-site models (where ω can vary both among amino acid sites in the protein and across branches on the tree). Specific details of the analyses are described below.

Likelihoods were obtained under the following site models: i) one ratio, i.e., the same ω for all sites (M0), ii) two classes of neutral sites in which either ω = 1 or ω = 0 (M1a), iii) positive selection, i.e., as for M1a but with a third class of sites under positive selection (ω > 1) (M2a), iv) sites follow a general discrete distribution (M3), v) ω follows a beta distribution (M7), vi) as for M7 but with an additional class of sites under positive selection (ω > 1) (M8). A complete description of the models is available in the PAML manual.

The six models were applied to concatenated mtDNA coding sequences. Likelihood ratio tests (LRT) were used to compare pairs of models. M0-M3 LRT was used to test for variable ω between sites. M1a-M2a LRT and M7-M8 LRT were both used to determine the existence of positively selected sites. Sites were considered to have experienced positive selection when at least one codon model showed ω > 1 and one LRT was significant for either the M1a-M2a or M7-M8 comparisons.

Posterior probabilities for site classes were obtained using a Bayes Empirical Bayesian (BEB) approach [30]. This allowed identification of positively selected sites under the M2a and M8 models. We considered that there was strong evidence for a coding site being under positive selection when the BEB posterior probability was greater than 0.9 and ω > 1. This represents a stringent requirement given that ω is unlikely to average more than 1 across the entire group.

In the widely-used branch-site model known as model A, the branches are divided into foreground and background groups [30]. (This model provided more conservative LRT results than those of the branch-based test model [31]). In the foreground lineages a proportion of the sites are under positive selection (ω > 1), while the background lineages feature a class of conserved sites with ω between 0 and 1, and a class of neutral sites with ω fixed at 1 [30]. Because it was difficult to select foreground branches a priori, we defined each possible branch as a foreground branch and then tested whether positive selection was present. Selection was tested by comparison of two models: null Model A, in which the sites in the foreground lineages are set at ω = 1, against the alternative Model A, in which ω > 1. We used the BEB to obtain posterior probabilities.

We conducted additional analyses to evaluate the robustness of the signal of positive selection in these data. The instantaneous rate change of codons *i* to *j* depends on parameter π_j_ (equilibrium frequency of codon *j*), which can be estimated in several ways: i) using nucleotide frequencies observed at each of the three codon positions within the gene (F3 × 4 model), ii) using the empirical nucleotide frequencies observed at the gene irrespective of codon position (F1 × 4) and iii) with all nucleotide frequencies are set equal (Fequal). Hence, we repeated the M7-M8 and Model A LRTs under the F1 × 4 and Fequal models for comparison with the F3 × 4 model.

### Analyses of physicochemical changes

Additional analyses of selection on amino acids were carried out using the program TreeSAAP ver. 3.2 software [32] which analyses significant changes in up to 31 physicochemical amino acid properties during evolution. The basis of this approach is comparison of changes in physicochemical properties inferred for the specified topology with an expected distribution of changes in under the condition that any amino acid is equally likely to be replaced by any other (as is the case under the neutral model). Congruent findings with the analyses of non-synonymous: synonymous ratios provide stronger evidence for selection. The mtDNA tree was used to infer amino acid replacement events and these were then assigned to one of eight physicochemical ‘magnitude of change’ categories (one being the smallest change and eight the highest). We focused only on physicochemical properties in categories 6, 7 and 8 as these represent the strongest positive selection. A sliding window size of 15 sites was used. Significance was assessed by comparing observed and expected mean changes in amino acid properties, χ^2^ goodness-of-fit tests were used to compare observed with expected distributions and deemed notably significant where *P* < 0.0001.

### Structure and co-evolutionary analyses of protein residues

Protein alignments containing amino acid residues were used to determine whether positively selected sites that were related to protein functions or located in important protein functional domains. Co-evolution between sites under positive selection and other sites within each protein was analysed using the online system at http://coevolution.gersteinlab.org/coevolution/ using Statistical Coupling Analysis (SCA). A total of 800 sequences were aligned (the maximum number allowed). These included the reference sequence and known structure (PDB ID 3RKO) of the ND protein within Complex I (only ND was analysed following the results of the selection analyses), one *P. putjatia* sequence from our study and 798 sequences from different genera from Uniprot online (mostly from the SWISS-PROT database). The sequence alignments corresponding to the five important ND subunits are given in the Additional file 3. Chimera X software [33] was used to portray the three-dimensional structure of proteins. The locations of *Phrynocephalus* residues found to be under selection within the PDB ID 3RKO structural model were displayed. In addition, 25 ND protein structures which encompassed the *Phrynocephalus* sites under selection were independently estimated using the Phyre2 online platform [34]. The residue side chains (for loci under selection) were obtained for all 19 individuals. To determine possible coevolutionary roles for a given selective residue in proton channel or other residues, Z-tests were computed on: i) SCA scores (s) (which do not incorporate distance between loci) ii) distances between loci (d) and iii) s/d [35–37].

## Results

### Mitochondrial genomes and phylogenetic analyses

Eight mitogenomes for the species/subspecies *P. v. pylzowi*, *P. v. vlangalii*, *P. t. theobaldi*, *P. frontalis*, *P. t. orientalis*, *P. e. erythrurus*, and *P. v. nanschanica* were obtained using PCR-based sequencing. Genome lengths varied from 16,251 bp (*P. v. nanschanica*) to 17,129 bp (*P. t. orientalis*). All sequences have been deposited in Genbank (GenBank accessions: MF039058–65).

The ML *Phrynocephalus* tree inferred from the mitogenomes was similar, but not identical, to that inferred previously [20] (Additional file 4). Topological differences involved nodes that were not strongly supported. In the current analysis, bootstrap support for the split between *P. forsythi* and the remaining *Phrynocephalus* was weak showing uncertainty over this relationship. Jin & Brown (2013) found strong (Bayesian) support for *P. forsythi* originating from the most basal viviparous node in both nuclear and mtDNA trees. Hence we favoured the latter *P. forsythii* position within the viviparous clade. The position of *P. helioscopus* within the oviparous group also differed from our previous Bayesian analysis. However, posterior support for the corresponding node was weak in that analysis, so the current ML topology for oviparous species was favoured. In sum, one adjustment was made to the topology of the ML tree for use in selection analyses.

### Random-sites analyses of selection

Values of ω much greater than 1 were detected for models M2A and M8 (both allow a proportion of sites to be under positive selection), while M3 did not reveal any positively selected sites (Table 1). The LRT that compared M0-M3 was highly significant (*P* < 0.001), which indicated a strong signal of variable ω among sites (Table 2). The M1a-M2a LRT was not significant (*P* > 0.9996) (Table 2). Nevertheless, the M7-M8 LRT was highly significant (*P* < 0.0001). These results are not unexpected as the M1a-M2a LRT has very low power compared with the M7-M8 LRT, particularly when the proportion of positively selected sites is very small [38]. Posterior probabilities obtained using BEB methods under M8 showed strong evidence of long-term positive selection at four sites within three ND subunits (Table 3): one site in *ND3* (#9), two sites in *ND4* (#187 and #454) and one site in *ND6* (#100).

**Table 1:** Site-models, log-likelihood values, transition: transversion ratio (κ) and parameter estimates for *Phrynocephalus* mitochondrial gene sequences. ω and ω_i_ are nonsynonymous: synonymous ratios, p_i_ refers to the proportion of sites in class *i* (see PAML manual for more details on these site models)

Model Number	Log Likelihood	κ	Parameter Estimates
M0 (one ratio)	−53,947.45	8.02	ω = 0.0755
M1A (nearly neutral)	−53,215.56	8.69	p_0_ = 0.9173, p_1_ = 0.0827 ω_0_ = 0.0410, ω_1_ = 1.0000
M2A (positive selection)	−53,215.56	8.69	p_0_ = 0.9173, p_1_ = 0.0827, p_2_ = 0.0000 ω_0_ = 0.0410, ω_1_ = 1.0000, ω_2_ = 30.1188
M3 (discrete)	−52,976.30	8.20	p_0_ = 0.7920, p_1_ = 0.0003, p_2_ = 0.2077 ω_0_ = 0.0149, ω_1_ = 0.0533, ω_2_ = 0.3429
M7 (beta)	−52,936.28	8.27	*p* = 0.1797, q = 1.7626
M8 (beta & ω)	−52,926.55	8.29	p_0_ = 0.9981, p_1_ = 0.0020, ω_s_ = 2.2162, *p* = 0.1863, q = 1.9069

**Table 2:** Results of log-likelihood ratio tests that compare different site models

Test for	Null Model	Experimental Model	df	*P*-value
Difference in ω among sites	Model 0 (one ratio)	Model 1A (nearly neutral)	2	< 0.0001
Positive selection	Model 1A (nearly neutral)	Model 2A (positive selection)	2	> 0.9996
Difference in ω among sites	Model 0 (one ratio)	Model 3 (discrete)	2	< 0.0001
Positive selection	Model 7 (beta)	Model 8 (beta & ω)	2	< 0.0001

**Table 3:** Sites found to be under long-term positive selection, as determined from posterior probabilities obtained using the Bayes empirical Bayes (BEB) approach

Gene Name	Amino Acid Position	BEB
*ND3*	9	0.988
*ND4*	187	0.987
*ND4*	454	0.988
*ND6*	100	0.994

**Fig. 2:**
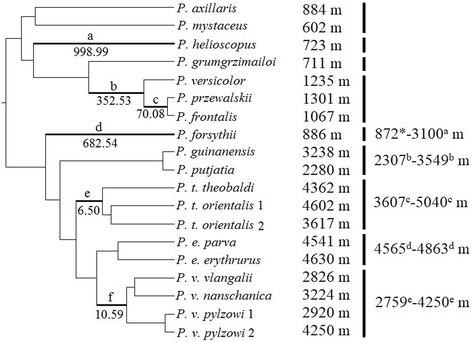
Phylogenetic tree that was used for selection analyses and showing branches on which there was evidence of selection: branches a, b, c, d, e, and f (bold lines) were found to have highly significant values of ω (non-synonymous: synonymous ratios), provided as branch labels. Altitudes of capture sites are given on the left of dashed lines, while the altitudinal ranges that have been described for the oviparous species are given on the right of these lines. References for these descriptions are denoted as *unpublished personal observation, ^a^ [51], ^b^ [52], ^c^ [53], ^d^ [54], ^e^ [55]

Branch-site analysis revealed several sites under episodes of positive selection on just six branches of the tree (Branches a-f in Fig. 2). Both the oviparous and viviparous groups contained three of these branches. Perhaps the most notable of these was the ancestral branch of all *P. theobaldi*, which reaches the highest elevation of all species. These corresponded to genes (site positions): *ND2* (#10), *COXIII* (#171), *ND4* (#109 and 448), *ND5* (#365) and *ND6* (#137) (Table 4). Multiple sites from different branches (> 90%) experienced purifying selection, approximately 8% of sites were neutral, while less than 1% of sites had undergone positive selection (Table 4).

**Table 4:** Parameter estimates (to 3 d.p.) under null and alternative hypotheses for branch-site Model A (four parameter version), for six significant branch/site combinations. Background site classes are: 0 (0 < ω_0_ < 1), 1 (ω_1_ = 1), 2a (0 < ω_0_ < 1) and 2b (ω_1_ = 1). Foreground site classes are: 0 (0 < ω_0_ < 1), 1 (ω_1_ = 1), 2a (ω_2_ ≥ 1) and 2b (ω_2_ ≥ 1). The six branches (a, b, c, d, e, f) are shown in Fig. 1

Branch	Amino Acid Position (gene)	Model	Parameter	0	1	2a	2b
a	109 (ND4)	Model A (Null)	Proportion	0.913	0.082	0.004	0.000
			Background ω	0.040	1.000	0.041	1.000
			Foreground ω	0.041	1.000	1.000	1.000
		Model A (Alternative)	Proportion	0.915	0.082	0.003	0.000
			Background ω	0.041	1.000	0.041	1.000
			Foreground ω	0.041	1.000	998.999	998.999
b	137 (ND6)	Model A (Null)	Proportion	0.917	0.083	0.001	0.000
			Background ω	0.041	1.000	0.041	1.000
			Foreground ω	0.041	1.000	1.000	1.000
		Model A (Alternative)	Proportion	0.917	0.083	0.001	0.000
			Background ω	0.041	1.000	0.041	1.000
			Foreground ω	0.041	1.000	352.529	352.529
c	10 (ND2)	Model A (Null)	Proportion	0.908	0.082	0.010	0.001
			Background ω	0.041	1.000	0.041	1.000
			Foreground ω	0.041	1.000	1.000	1.000
		Model A (Alternative)	Proportion	0.917	0.082	0.000	0.000
			Background ω	0.041	1.000	0.041	1.000
			Foreground ω	0.041	1.000	70.079	70.079
d	448 (ND4)	Model A (Null)	Proportion	0.914	0.083	0.003	0.000
			Background ω	0.041	1.000	0.041	1.000
			Foreground ω	0.041	1.000	1.000	1.000
		Model A (Alternative)	Proportion	0.917	0.083	0.000	0.000
			Background ω	0.041	1.000	0.041	1.000
			Foreground ω	0.041	1.000	682.538	682.538
e	171 (COXIII)	Model A (Null)	Proportion	0.884	0.080	0.034	0.003
			Background ω	0.041	1.000	0.041	1.000
			Foreground ω	0.041	1.000	1.000	1.000
		Model A (Alternative)	Proportion	0.911	0.082	0.006	0.001
			Background ω	0.041	1.000	0.041	1.000
			Foreground ω	0.041	1.000	6.409	6.409
f	365 (ND5)	Model A (Null)	Proportion	0.909	0.082	0.008	0.001
			Background ω	0.041	1.000	0.041	1.000
			Foreground ω	0.041	1.000	1.000	1.000
		Model A (Alternative)	Proportion	0.916	0.083	0.001	0.000
			Background ω	0.041	1.000	0.041	1.000
			Foreground ω	0.041	1.000	10.593	10.593

Re-analyses of the M7-M8 LRT and Model A LRT using the F1 × 4 and Fequal models on individual coding genes rather than the concatenated sequence revealed the same positively selected sites and similar posterior probabilities. This supported the robustness of both the site and branch-site analyses.

### Analyses of physicochemical changes

**Fig. 3:**
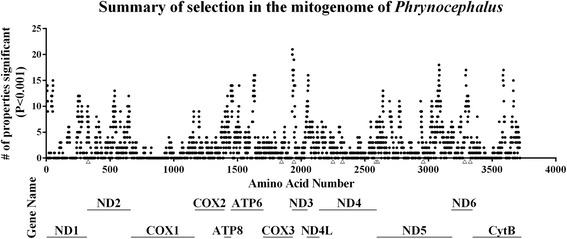
Summary of selection in the mitogenome of *Phrynocephalus*. The 13 mitochondrial coding genes are represented and labelled (figure does not include tRNAs). Filled black circles represent positively selected sites (*p* < 0.001) identified by TreeSAAP analysis. The dataset was analysed for significant changes in each of the 31 physicochemical properties. The y-axis represents the number of properties for which selection was inferred (for each site). Open triangles are positively selected sites identified by independent analyses of ω

More than half (sixteen) of the eigenvalues for physicochemical characteristics changed in selective sites within *ND3* (#9) and *ND6* (#100), both of which had experienced long-term positive selection. Moreover, all other residues except *COXIII* (#171) that showed evidence of selection (in PAML analyses) corresponded to the greatest observed changes in physicochemical characteristics within each subunit (see Fig. 3). One ND1 site showed changes in physicochemical properties, eleven of which represented changes to basic characteristics of the subunit, despite significant positive selection not previously being detected. ND subunits under selection, as detected by analyses of ω, therefore also tended to show important physicochemical changes.

### Structural and co-evolutionary analyses of protein amino acid residues

**Fig. 4:**
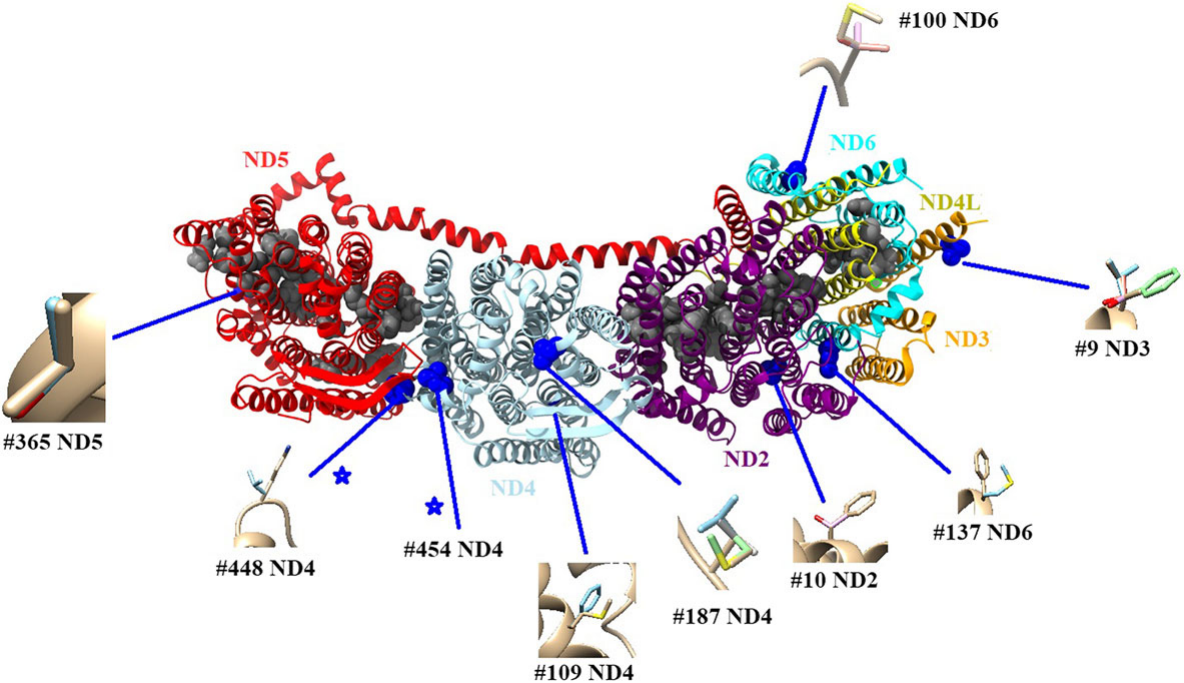
The three dimensional structure of Complex I showing the locations of amino acids under selection as determined by predictive models for individual *Phrynocephalus* ND subunits. Residues marked as blue circles are the selected residues within the reference crystal structure (PDB ID 3RKO). Diagrams of residue side chains are given for each. Each ND subunit is coloured individually, protein names correspond to the protein subunits in Complex I. Blue stars indicate the two residues that show significant co-evolutionary relationship with key residues (in grey sphere) in the chain of proton translocation

Nine out of the ten sites that were detected by the site and branch-site model analyses were in ND subunits and therefore provided strong evidence of selection primarily affecting the hydrophobic region of Complex I of the mitochondrial respiratory chain (Fig. 4), the remaining site was in Complex III. All selected sites in ND subunits were located in the helices of these subunits in the Complex I crystal structure (Fig. 4), while two residues (#187 ND4, #100 ND6) appeared in random coils but quite near the residues of helices in the simulated 3D structure (high confidence was obtained for all, i.e., 100%). The final residue (#454) of ND4 was absent in the template and could not be included. The simulated three-dimensional structure (Fig. 4) shows that changes at all of the selected residues caused structural changes in residue side chains (Fig. 4).

SCA analyses of co-evolution of protein spatial structure (the hydrophobic region of Complex I) provided probabilities close to the 0.05 level (*P* = 0.049 and *P* = 0.060, respectively) for each of the two selected residues in ND4 (#454 and #448). This provided some evidence for co-evolutionary relationships with residues that played key or important roles in proton transport as well as other residues not responsible for proton translocation (Table 5).

**Table 5:** Results of SCA co-evolutionary analyses for each selective site. The *t* and corresponding *P-values* are given for the SCA scores (s), distances between sites (d), and the ratios of these measures (s/d)

	Selective sites	*n*	s		d		s/d	
Gene			*t*	*P*	*t*	*P*	*t*	*P*
ND2	10	346	0.430	0.670	−2.065	0.045*	1.140	0.255
ND3	9	91	0.774	0.513	0.171	0.877	1.058	0.293
ND4	109	421	1.344	0.192	−0.554	0.584	0.137	0.891
ND4	187	421	0.285	0.778	1.042	0.305	0.973	0.331
ND4	448	421	1.422	0.168	−1.618	0.118	1.851	0.062
ND4	454	421	−1.743	0.093	−0.949	0.351	−1.967	0.049*
ND5	365	537	1.004	0.333	−4.434	< 0.001*	−0.015	0.988
ND6	100	97	−0.552	0.598	−2.795	0.027*	0.715	0.496
ND6	137	97	1.715	0.128	−0.341	0.743	1.488	0.143

*P < 0.05

## Discussion

The hydrophobic region of the respiratory Complex I comprises mitochondrial proteins: one piston arm of its sub-element NuoL (equates to ND5) combines with three proton pumps including NuoL (ND5), NuoM (ND4) and NuoN (ND2). This structure plays an important role in proton transport for ATP synthesis [39]. A total of nine out of ten selective sites were related to ND proteins within Complex I and indicated that there was stronger selection on ND genes than on mitochondrial genes that coded for other respiratory chain complexes*.* The positively selected sites were not key sites directly responsible for proton translocation in the channel, but at least two selected sites in NuoM (ND4) do appear to have a coevolutionary relationship with proton transport channel residues.

In addition to detection of Complex I selection through non-synonymous: synonymous ratios, we found notable changes in the selected residues’ physicochemical properties. Sites with highest nonsynonymous: synonymous ratios tended to show some of the greatest changes in physicochemical properties suggesting that selection was acting at sites associated with important structures/functions.

Several studies have used variation in non-synonymous: synonymous ratios to infer variable selection pressures across mitochondrial genes [40] including ND subunits [7, 41–45]. The degree of hydrophobicity has been found to differ among animal groups for the ND5 subunit, and to a lesser extent the ND1, ND2 and ND4 subunits, pointing towards possible functions associated with selection [44]. Four out of nine selected sites in Complex I were found in *ND4*, indicating that this subunit might experience particularly strong selection in response to environmental differences. While several studies have found selection on Complex I, e.g., [7, 17], others have found selection on other complexes, e.g., [19]. For example, research on primates has indicated that electron transport chain-related proteins of mitochondrial Complex III and Complex IV underwent distinct selection pressures [9].

Adaptive responses to high-altitude in vertebrates are likely to involve numerous genetic interactions. How differences in elevation might shape selection on the ND complex in ectotherms remains speculative. *Phrynocephalus* lizards living at high altitudes/latitudes need to store and release energy to fuel metabolism under low oxygen concentrations and/or at lower temperatures than species from warmer regions. Several temperature-dependent traits, such as locomotor performance, are likely to be under quite strong selection pressures in lizards [46] and are linked to oxidative phosphorylation. Colonization of lower temperature environments might therefore be expected to lead to selection on this process, involving changes in the associated proteins. The lack of clear statistical evidence to support positive selection in the ancestor of all viviparous lineage therefore runs counter to this expectation because this lineage is potentially associated with the largest change in altitude. Nevertheless, there are some more subtle indications of altitude-related selection on Complex I, which are discussed below.

Proton transport is closely related to mitochondrial metabolism. Previous analyses of positive selection on protein-coding genes have often been linked to external factors, such as climatic variation [16, 47, 48], high temperature and physical activity [49], extreme temperatures and oxygen levels [50]. Mitochondrial respiratory rate appears to differ in *P. erythrurus* relative to a congeneric oviparous species, providing potential advantages at high altitude [25]. Our branch site analyses indicated that three lineages within the high-elevation viviparous group, namely *P. vlangalii*, *P. forsythii* and *P. theobaldi*, had undergone significant positive selection during the evolutionary history of the group. *Phrynocephalus theobaldi* reaches some of the highest elevations within this group which could explain why selection was detected in the lineage ancestral to the both of the subspecies analysed. *Phrynocephalus forsythii* inhabits a wide elevational range and extends down to the lowest ranges within the group (in fact, the capture site for *P. forsythii* here was much lower than the known elevational ranges of other viviparous *Phrynocephalus* lizards, by approximately 2000-4000 m). Similarly we detected positive selection in the *P. vlangalii* group, which was found at lower elevations than the sister taxa. Hence the latter two species do not exhibit selection for high altitudes, but potentially the opposite (selection for low altitudes). In the oviparous group, the branch that is ancestral to *P. versicolor*, *P. przewalskii*, and *P. frontalis*, and a subsequent branch ancestral to the latter two species, both show evidence of selection. These species are found at higher altitudes than other oviparous species and are distributed in the cooler eastern highlands, suggesting temperature related positive selection. The branch leading to *P. helioscopus* also show evidence of very strong selection. While this is found at lower altitudes than the *P. versicolor*, *P. przewalskii*, and *P. frontalis* group, it has the northernmost distribution of all *Phrynocephalus* lizards which again might suggest temperature-related selection.

Our research overcame some limitations of previous studies. For example, it is often relatively difficult to specifically detect sites that have experienced selection and are of functional significance because: i) most previous studies only analysed subsets of codons/mitochondrial protein-coding regions, ii) species groups used in some studies were incomplete, and so branches represented longer and potentially more varied histories; iii) unlike many previous studies, we were able to analyse mitochondrial protein-coding regions that corresponded to well-known structures and functional sites (specifically Complex I).

## Conclusions

Taken together, we found strong evidence for selection on subunits associated with the hydrophobic region of mitochondrial complex I and we infer that this is at least partially associated with historical altitude and latitude range shifts that individual species within the group have undergone. The decrease in environmental temperatures with increasing altitude and latitude may have led to selection on oxidative phosphorylation, but consideration of the adaptive nature of these molecular changes remains speculative. Future studies of climate-related selection on the protein complexes associated with oxidative phosphorylation in ectotherms will help to determine general trends and allow more detailed insights into the processes involved.

## Additional files

Additional file 1: Voucher specimen and mitogenome used in selection analyses. (DOC 49 kb)

Additional file 2: Primer pairs used in amplification of fragments of mitogenomes. (DOC 63 kb)

Additional file 3: Multiple sequence alignment was used in statistical coupling analyses for ND proteins. (XLS 224 kb)

Additional file 4: ML tree for *Phrynocephalus* inferred from 15,417 bp of mtDNA (6 partitions; GTRGAMMA model for each). Values on nodes are bootstrap proportions (2000 bootstraps). (DOCX 172 kb)
