# Supplementary material for: Evolutionary analysis of mitochondrially encoded proteins of toad-headed lizards, Phrynocephalus, along an altitudinal gradient
Source: BMC Genomics. 2018 Mar 6;19:185. doi: 10.1186/s12864-018-4569-1 (PMC5840783; doi:10.1186/s12864-018-4569-1)
Supplement: Supplementary file 1 — Voucher specimen and mitogenome used in selection analyses. (DOC 49 kb) [file 12864_2018_4569_MOESM1_ESM.doc]

Online Supplementary Information S1. Voucher specimen and mitogenome used in selection analyses.

| Taxon | Voucher specimen | Longitude | Latitude | Altitude (m) | GenBank Acc. | Locality |
| --- | --- | --- | --- | --- | --- | --- |
| *P. v. pylzowi* 1 | MQ | 102.90E | 34.96N | 2920 | MF039059 | Maqu County, northeastern Qinghai-Tibetan Plateau (QTP), |
| *P. t. orientalis* 2 | PT4003 | 90.83E | 29.38N | 3617 | KJ551842 | Qushui County, Tibet |
| *P. guinanensis* | GN | 100.63E | 35.66N | 3238 | KJ885621 | Sand dunes, Guinan County, Qinghai Province |
| *P. v. pylzowi* 2 | MD | 98.06E | 34.44N | 4250 | MF039060 | Madoi County, located upstream of the Yellow River, northeastern Qinghai- Tibetan Plateau (QTP) |
| *P. putjatia* | GD | 101.44E | 36.02N | 2280 | KJ830752 | Nanhai temple in Guide County, Qinghai Province |
| *P. v. vlangalii* | GEM | 95.03E | 36.39N | 2826 | MF039058 | Geermu, Western Qinghai-Xizang (Tibetan) Plateau |
| *P. versicolor* | GZ | 95.70E | 40.65N | 1235 | KJ749841 | Guazhou County, Gansu Province |
| *P. e. parva* | PEP | 92.26E | 34.93N | 4541 | KJ630904 | Tuotuo River region, Northern Qiangtang Plateau of Tibet |
| *P. mystaceus* | PM | 80.79E | 44.02N | 601.8 | KC578685 | Tukai Desert, Huocheng County |
| *P. przewalskii* | PRZ | 105.02E | 37.47N | 1301 | KF572032 | Shapotou, Zhongwei City, Ningxia Hui Autonomous Region |
| *P. axillaris* | RQ | 88.03E | 38.98N | 884 | KC119493 | Ruoqiang County, Xinjiang Uygur Autonomous Region |
| *P. t. theobaldi* | PT2605 | 80.25E | 33.12N | 4362 | MF039063 | Ngari Prefecture, Tibet |
| *P. grumgrzimailoi* | PGR | 90.12E | 44.34N | 711 | KM093859 | Qitai County, Xinjiang Uygur Autonomous Region |
| *P. helioscopus* | PH | 86.06E | 46.45N | 723 | KM093858 | Hoxtolgay Town, Mongolian Autonomous County of Hoboksar, Xinjiang Uygur Autonomous Region |
| *P. frontalis* | WLT | 108.72E | 40.75N | 1067 | MF039064 | Urad Front Banner, Inner Mongolia |
| *P. t. orientalis* 1 | PT3103 | 83.49E | 30.03N | 4602 | MF039062 | Brahmaputra River valley, Tibet |
| *P. e. erythrurus* | PEE | 91.58E | 32.15N | 4630 | MF039065 | Amdo, Southern Qiangtang Plateau of Qinghai-Tibetan Plateau |
| *P. v. nanschanica* | PVN | 96.06E | 38.97N | 3224 | MF039061 | Nanshan Mountains, Southern Danghe range, Northern Qinghai-Tibetan Plateau |
| *P. forsythii* | FOR | 86.17E | 41.39N | 886 | KP126516 | Yuli County, Xinjiang Uygur Autonomous Region |
