# Supplementary material for: Evolutionary analysis of mitochondrially encoded proteins of toad-headed lizards, Phrynocephalus, along an altitudinal gradient
Source: BMC Genomics. 2018 Mar 6;19:185. doi: 10.1186/s12864-018-4569-1 (PMC5840783; doi:10.1186/s12864-018-4569-1)
Supplement: Supplementary file 2 — Primer pairs used in amplification of fragments of mitogenomes. (DOC 63 kb) [file 12864_2018_4569_MOESM2_ESM.doc]

Online Supplementary Information S2. Primer pairs used in amplification of fragments of mitogenomes.

| **Fragments** | **Primers** | **Length of amplified fragments** | **Species/distribution** |
| --- | --- | --- | --- |
| *Cytb-12S rRNA*  *12S rRNA-ND2*  *ND2-ND4*  *ND4-Cytb*  *C*ontrol region  *Cytb-12S rRNA*  *12S rRNA-ND2*  *ND2-ND4*  *ND4-Cytb*  *C*ontrol region  *Cytb-12S rRNA*  *12S rRNA-ND2*  *ND2-ND4*  *ND4-Cytb*  *C*ontrol region  *Cytb-12S rRNA*  *12S rRNA-ND2*  *ND2-ND4*  *ND4-Cytb*  *C*ontrol region  *Cytb-12S rRNA*  *12S rRNA-ND2*  *ND2-ND4*  *ND4-Cytb*  *C*ontrol region  *Cytb-12S rRNA*  *12S rRNA-ND2*  *ND2-ND4*  *ND4-Cytb*  *C*ontrol region  *Cytb-12S rRNA*  *12S rRNA-ND2*  *ND2-ND4*  *ND4-Cytb*  *C*ontrol region  *Cytb-12S rRNA*  *12S rRNA-ND2*  *ND2-ND4*  *ND4-Cytb*  *C*ontrol region  *Cytb-12S rRNA*  *12S rRNA-ND2*  *ND2-ND4*  *ND4-Cytb*  *C*ontrol region  *Cytb-12S rRNA*  *12S rRNA-ND2*  *ND2-ND4*  *ND4-Cytb*  *C*ontrol region  *Cytb-12S rRNA*  *12S rRNA-ND2*  *ND2-ND4*  *ND4-Cytb*  *C*ontrol region  *Cytb-12S rRNA*  *12S rRNA-ND2*  *ND2-ND4*  *ND4-Cytb*  *C*ontrol region  *Cytb-12S rRNA*  *12S rRNA-ND2*  *ND2-ND4*  *ND4-Cytb*  *C*ontrol region  *Cytb-12S rRNA*  *12S rRNA-ND2*  *ND2-ND4*  *ND4-Cytb*  *C*ontrol region  *Cytb-12S rRNA*  *12S rRNA-ND2*  *ND2-ND4*  *ND4-Cytb*  *C*ontrol region  *Cytb-12S rRNA*  *12S rRNA-ND2*  *ND2-ND4*  *ND4-Cytb*  *C*ontrol region | Forward: CGCCGTCGACAGCCCATTTT  Reverse: GAAGTGAATTGGGGCAGCTC  Forward: TGGGCCCATGCCCCAAAAAC  Reverse: AGGTTAGGCCATGGGCAATT  Forward: CGCATAACCCCAACAAACA  Reverse: GATGCTCCGTTTGCATGAAT  Forward: ATCTTTTTAGCCGCTCACTTT  Reverse: GACTGGCTGGACAATGATGC  Forward: CTTCATATGCTCAAGCGGAGCGATATCTT  Reverse: AATCTCGAGGTAGGCGTGGTTTCACAGGA  Forward: CGCCGTCGACAGCCCATTTT  Reverse: GAAGTGAGTTGGGGCAGCTC  Forward: TGGGTTCATGCCCCAAAAAC  Reverse: AGGTTAAGCCGTGGGCAACT  Forward: TCGCATAACCCCAATAAACA  Reverse: GATGCTCCGTTTGCATGAAT  Forward: ATCTTTTTAGCCGCTCACTTT  Reverse: GACTGGCTGGACAATGATGC  Forward: CTTCATATGCCCAAGCGGAGCGATATCTT  Reverse: AATCTCGAGGTAGGCGTGGTTTCACAGGA  Forward: CGCCGTCGACAGCCCATTTT  Reverse: GAAGTGAACTGGGGCAGCCC  Forward: TGGGCCCATGCCCCAAAAAC  Reverse: AAGTTAAGCCATGGGCGACT  Forward: CCGCATATCCCCAACAAACC  Reverse: GATGCTCCATTTGCATGAAT  Forward: ATCTTTTTAGCCGCTCACTTT  Reverse: GACTGGCTGGACAATGATGC  Forward: CTTCATATGCTCAAGCGGAGCGATATCTT  Reverse: AATCTCGAGTGTAGGCGTATTTCACTGGA  Forward: CGCCGTCGACAGCCCATTTT  Reverse: GAAGTGAATTGGGGCAGCTC  Forward: TGGGCCCATGCCCCAAAAAC  Reverse: AGGTTAGGCCATGGGCAATT  Forward: CCGCATAACCCCAACAAACA  Reverse: GATGCTCCGTTTGCATGAAT  Forward: ATCTTTTTAGCCGCTCACTTT  Reverse: GACTGGCTGGACAATGATGC  Forward: CTTCATATGCTCAAGCGGAGCGATATCTT  Reverse: AATCTCGAGTGTAGGCATGTTTCACGGGG  Forward: CGCCGTCGACAGCCCATTTT  Reverse: GAAGTGAACTGGGGCAGCCC  Forward: TGGGCCCATGCCCCAAAAAC  Reverse: AAGTTAAGCCATGGGCGACT  Forward: CCGCATATCCCCAACAAACC  Reverse: GATGCTCCATTTGCATGGAT  Forward: ATCTTTTTAGCCGCTCACTTT  Reverse: GACTGGCTGGACAATGATGC  Forward: CTTCATATGCTCAAGCGGAGCGATATCTT  Reverse: AATCTCGAGTGTAGGCGTATTTCACTGGA  Forward: CGCCGTCGACAGCCCATTTT  Reverse: GAAGTGAATTGGGGCAGCTC  Forward: TGGGCCCATGCCCCAAAAAC  Reverse: AGGTTAGGCCATGGGCAATT  Forward: CCGCATAACCCCAACAAACA  Reverse: GATGCTCCGTTTGCATGAAT  Forward: ATCTTTTTAGCCGCTCACTTT  Reverse: GACTGGCTGGACAATGATGC  Forward: CTTCATATGCTCAAGCGGAGCGATATCTT  Reverse: AATCTCGAGTGTAGGCATGTTTCACGGGG  Forward: CGCCGTCGACAGCCCATTTT  Reverse: GAAGTGAATTGGGGCGGCCC  Forward: TGGGCCCATGCCCCAAAAAC  Reverse: AGGTTAAGCCGTGGGCAACT  Forward: CCGTATAACTCCAACAAACA  Reverse: GATGCCCCATTTGCATGAAT  Forward: ATTTTTTTAGCCGCTCACTTC  Reverse: GACTGGCTGGACAATGATGC  Forward: CTTCATATGCCCAAGCGGAGCGATATCTT  Reverse: AATCTCGAGTATAGGCGTATTTCACGGGA  Forward: CGCCGTCGACAGCCCATTTT  Reverse: GAAGTGAACTGGGGCAGCTC  Forward: TGGGCCCATGCCCCAAAAAC  Reverse: AGGTTAAGCCGTGAGCAACT  Forward: CCGCATATCTCCAACAAACA  Reverse: GATGCTCCGTTTGCATGAAT  Forward: ATCTTTTTAGCCGCTCACTTT  Reverse: GACTAGCTGGACAATGATGC  Forward: CTTCATATGCCCAAGCGGAGCGATATCTT  Reverse: AATCTCGAGTGTAGGCATGTTTCACGGGA  Forward: CGCCGTCGACAGCCCATTTT  Reverse: GAAGTGGATTGGAGCTGCCC  Forward: TGGGCCCATGCCCCAAAAAC  Reverse: AGGTTAAGCCGTGAGCAACT  Forward: CCGTATAACCCCAACAAACA  Reverse: GATGCCCCATTTGCATGGAT  Forward: ATTTTTTTAGCCGCTCACTTC  Reverse: GACTGGCTGGACAATGATGC  Forward: CTTCATATGCCCAAGCGGAGCGATATCTT  Reverse: AATCTCGAGTATAGGCGTATTTCACGGGA  Forward: CGCCGTCGACAGCCTATTTT  Reverse: GAAGTGAATTGGGGCTGCTC  Forward: TGGGCCCATGCCCCAAAAAC  Reverse: AGGTTAGGCCGTGGGAGACT  Forward: CCGCATAACCCCAACAATCA  Reverse: GATGCTCCGTTTGCATGAAT  Forward: ATCTTTTTAGCCACTCACTTT  Reverse: GACTGGCTGGACAATGATGC  Forward: CTTCATATGCCCAAGCGGAGCGATATCTT  Reverse: AATCTCGAGGTAGGTAAGGTTTAACGTGG  Forward: CGCCGTCGACAGCCCATTTT  Reverse: GAAGTGAATTGGGGCGGCCC  Forward: TGGGCCCATGCCCCAAAAAC  Reverse: AAGTTAAGCCGTGGGCAACT  Forward: CCGCATAACCCCAACAAACA  Reverse: GATGCTCCGTTTGCATGAAT  Forward: ATTTTTTTAGCCGCTCACTTT  Reverse: GACTGGCTGGACAATGATGC  Forward: CTTCATATGCCCAGGCGGAGCGATATCTT  Reverse: AATCTCGAGTATAGGCGAATTTCACGGGA  Forward: CGCCGTCGACAGCCCATTTT  Reverse: AAAGTGGATAGGGGCGGCTC  Forward: TGGGCCCATGCCCCAAAAAC  Reverse: AAGTTAGGCCGTGGGCGATT  Forward: TCGTATAGCCCCAACAAACA  Reverse: GATGCTCCGTTTGCATGAAT  Forward: GTCTTTTTAGCCGCCCATTTT  Reverse: GGCTGGCTAGACGATGATGC  Forward: CTTCATATGCCCAAGCGGAGCGATATCTT  Reverse: AATCTCGAGTATAGGCGTATTTCACGGGA  Forward: CGCCGTCGACAGCCCATTTT  Reverse: GAAGTGGATTGGAGCTGCCC  Forward: TGGGCCCATGCCCCAAAAAC  Reverse: AGGTTAAGCCGTGAGCAACT  Forward: CCGTATAACTCCAACAAACA  Reverse: GATGCCCCATTTGCATGGAT  Forward: ATTTTTTTAGCCGCTCACTTC  Reverse: GACTGGCTGGACAATGATGC  Forward: CTTCATATGCCCAAGCGGAGCGATATCTT  Reverse: AATCTCGAGTATAGGCGTATTTCACGGGA  Forward: CGCCGTCGACAGCCCATTTT  Reverse: GAAGTGAGTTGGGGCGGCTC  Forward: TGGGCCCATGCCCCAAAAAC  Reverse: AGGTTAGGCCATGGGCAACT  Forward: CCGCATAACCCCAACAAACA  Reverse: GATGCTCCGTTTGCATGAAT  Forward: ATCTTTTTAGCCGCCCACTTT  Reverse: GACTGGCTGGACAATGATGC  Forward: CTTCATATGCCCAAGCGGAGCGATATCTT  Reverse: AATCTCGAGGTAGGTAAGGTTTAACGTGG  Forward: CGCCGTCGACAGCCCATTTT  Reverse: GAAGTGAATTGGGGCAACTC  Forward: TGGGCCCATGCCCCAAAAAC  Reverse: AGGTTAAGCCATGAGCAATC  Forward: CCGCATATCTCCAACAAACA  Reverse: GATGCTCCGTTTGCATGGAT  Forward: ATCTTTTTAGCCGCTCACTTT  Reverse: GACTGGCTGGACAATGATGC  Forward: CTTCATATGCCCAAGCGGAGCGATATCTT  Reverse: AATCTCGAGTGTAGGCATGATTTCACGGG  Forward: CGCCGTCGACAGCCCATTTT  Reverse: GAAGTGAATTGGGGCAGCTC  Forward: TGGGCCCATGCCCCAAAAAC  Reverse: AGGTTAGGCCATGGGCAATT  Forward: CCGTATAACCCCAACAAACA  Reverse: GATGCTCCGTTTGCATGAAT  Forward: ATCTTTTTAGCCGCTCACTTT  Reverse: GACTGGCTGGACAATGATGC  Forward: CTTCATATGCTCAAGCGGAGCGATATCTT  Reverse: AATCTCGAGTGTAGGCATGTTTCACGGGG | 3423  7195  3400  3709  1348  3418  7196  3401  3872  869  4580  7193  3401  3545  901  3424  7196  3400  3989  1341  3423  7193  3401  3550  908  3422  7198  3400  3583  933  3421  7204  3401  3684  627  3422  7194  3397  3650  997  3421  7208  3401  4146  695  3419  7200  3402  3626  1069  3423  7200  3404  3558  892  3424  7198  3404  3504  753  3421  7208  3401  4295  846  3417  7197  3401  4395  1828  3420  7195  3400  3725  1074  3421  7195  3401  3516  868 | *P. v. pylzowi*  / Qinghai Plateau  *P. t. orientalis*  */* Southern Xizang Plateau  *P. guinanensis*  */* sand dunes in Guinan basin or valleys  *P. v. pylzowi*  */* Qinghai Plateau  *P. putjatia*  / Guide Basin or valleys  *P. v. vlangalii*  */* Qinghai Plateau  *P. versicolor*  */* mainly in Inner Mongolia Plateau  *P. e. parva*  / Northern Qianghai Plateau  *P. przewalskii*  */* mainly in Inner Mongolia Plateau  *P. t. theobaldi*  */* Southern Xizang Plateau  *P. grumgrzimailoi*  */* Eastern Zungeer Basin  *P. helioscopus*  */* Northern Zungeer Basin  *P. frontalis*  */* Inner Mongolia Plateau  *P. t. orientalis*  */* Southern Xizang Plateau  *P. e. erythrurus*  */* Southern Qiangtang Plateau  *P. v. nanschanica*  */* Nanshan area in Gansu, near Qinghai Plateau |
