# Supplementary material for: Evolutionary analysis of mitochondrially encoded proteins of toad-headed lizards, Phrynocephalus, along an altitudinal gradient
Source: BMC Genomics. 2018 Mar 6;19:185. doi: 10.1186/s12864-018-4569-1 (PMC5840783; doi:10.1186/s12864-018-4569-1)

Online Supplementary Information S4. ML tree for *Phrynocephalus* inferred from 15417bp of mtDNA (6 partitions; GTRGAMMA model for each). Values on nodes are bootstrap proportions (2000 bootstraps).


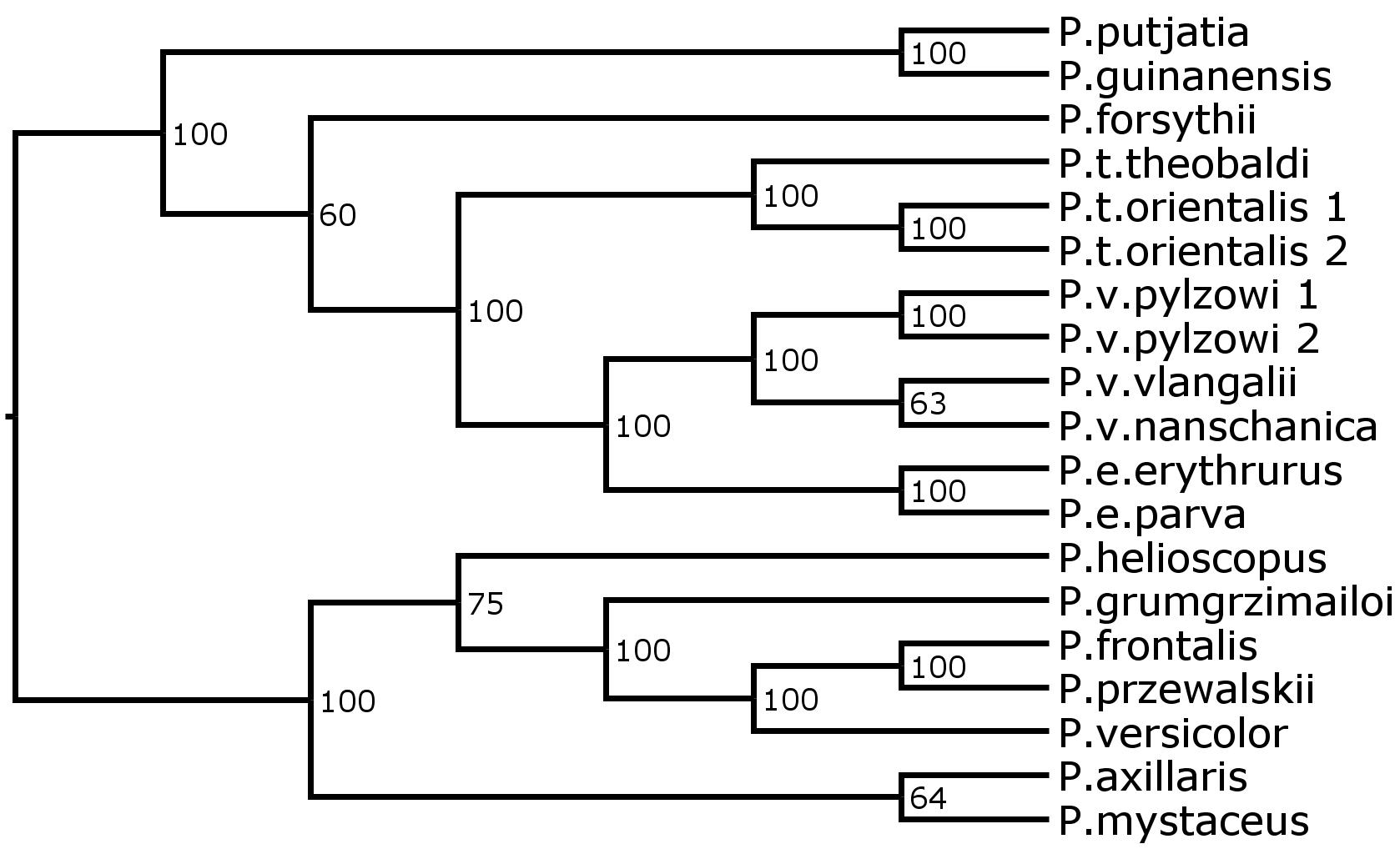

Supplement: Supplementary file 4 — ML tree for Phrynocephalus inferred from 15,417 bp of mtDNA (6 partitions; GTRGAMMA model for each). Values on nodes are bootstrap proportions (2000 bootstraps). (DOCX 172 kb) [file 12864_2018_4569_MOESM4_ESM.docx]
